# Patient-conducted anodal transcranial direct current stimulation of the motor cortex alleviates pain in trigeminal neuralgia

**DOI:** 10.1186/1129-2377-15-78

**Published:** 2014-11-25

**Authors:** Tim Hagenacker, Vera Bude, Steffen Naegel, Dagny Holle, Zaza Katsarava, Hans-Christoph Diener, Mark Obermann

**Affiliations:** 1 Department of Neurology, University Hospital Essen, Hufelandstr. 55, 45147 Essen, Germany

**Keywords:** Trigeminal neuralgia, Pain processing, Transcranial direct current stimulation, Clinical electrophysiology

## Abstract

**Background:**

Transcranial direct current stimulation (tDCS) of the primary motor cortex has been shown to modulate pain and trigeminal nociceptive processing.

**Methods:**

Ten patients with classical trigeminal neuralgia (TN) were stimulated daily for 20 minutes over two weeks using anodal (1 mA) or sham tDCS over the primary motor cortex (M1) in a randomized double-blind cross-over design. Primary outcome variable was pain intensity on a verbal rating scale (VRS 0–10). VRS and attack frequency were assessed for one month before, during and after tDCS. The impact on trigeminal pain processing was assessed with pain-related evoked potentials (PREP) and the nociceptive blink reflex (nBR) following electrical stimulation on both sides of the forehead before and after tDCS.

**Results:**

Anodal tDCS reduced pain intensity significantly after two weeks of treatment. The attack frequency reduction was not significant. PREP showed an increased N2 latency and decreased peak-to-peak amplitude after anodal tDCS. No severe adverse events were reported.

**Conclusion:**

Anodal tDCS over two weeks ameliorates intensity of pain in TN. It may become a valuable treatment option for patients unresponsive to conventional treatment.

## Background

Several studies showed that transcranial direct current stimulation (tDCS) modulates the cortical excitability of the motor cortex depending on the direction of the electric current (e.g. anodal or cathodal) and that its neuroplastic effects sustain after stimulation [1]. The primary effect of tDCS is a modulation of the membrane potential of neurons in the stimulated cortical area [2,3], mediated by N-methyl-D-aspartate receptors (NMDA-R) [4]. Nonetheless, the effects of tDCS on cortical excitability can spread to distant cortical areas possibly along interconnections between the stimulated area and adjacent regions [5]. It was suggested, that anodal tDCS modulates pain perception by shifts of the resting membrane potential and consequent alteration of the corticospinal excitability at the stimulation site. tDCS was demonstrated to improve pain symptoms in patients with different types of chronic pain [6] but with conflicting results. While in painful diabetic neuropathy anodal tDCS produces pain relief, in chronic lower back pain and postoperative pain the results were conflicting, while cathodal nor anodal tDCS was ineffective [7-9], recently a combination of tDCS and peripheral electrical stimulation has been shown to improve low back pain symptoms more effectively than either applied alone or a sham control [10]. In chronic headaches, anodal tDCS was demonstrated to be an effective non-invasive treatment, but for classical trigeminal neuralgia (TN) and orofacial pain data is rare [11].

TN is a rare facial pain disorder with a prevalence of 0.1 – 0.2/1000 that leads to paroxysms of short lasting but very severe pain [12]. In most cases the third and second branch of the trigeminal nerve are affected [13]. Pain can occur spontaneously but also triggered attacks due to e.g. eating, talking or brushing the teeth occur frequently [13]. Between the attacks the patient is usually asymptomatic, but a constant dull background pain in the affected trigeminal facial area may persist [13], which has been suggested to be a sign of central sensitization and chronification of pain [14]. Carbamazepine is currently the drug of first choice in the treatment of TN. It is effective in 70-80% of patients but often associated with severe adverse effects such as drowsiness, confusion, nausea and ataxia, which may require discontinuation of medication [15]. TN often begins with higher age, which lowers the tolerability of side effects and interactions due to medication [16]. Surgical interventions, like the microvascular decompression, stereotactic radiotherapy or the percutaneous may not be suitable for all patients and waiting for the procedure can be agonizing. Therefore, different non-invasive treatment options are indispensable. This study aims to investigate the efficacy of anodal tDCS of the motor cortex in the treatment of TN with and without concomitant permanent pain using a randomized, cross-over design. Pain evoked potentials and the nociceptive blink reflex were used as objective measure of the tDCS effect on human trigeminal pain processing.

## Methods

### Patients

Seventeen patients were enrolled in the study. Five patients finished anodal stimulation and discontinued the study without sham stimulation, one patient discontinued after sham stimulation without anodal treatment and one patient did not stimulate at all as recorded in the stimulator. Three of the patients that were only treated anodal, reported the application of the stimulation as too difficult, one patient reported an increase of attacks and one patient reported no further interest in the stimulation. In total, 10 patients completed the study with a mean age of 63 years (range: 49–82 years; 4 patients started with anodal treatment, 6 with sham treatment; see Table 1). Inclusion criteria were: classical TN with or without concomitant permanent pain according to the beta-version of the 3rd edition of the International Classification of Headache Disorders (ICHD-3 beta, chapter 13.1.1). Six patients suffered from classical TN with concomitant permanent pain (ICHD-3 beta chapter 13.1.1.2), four were included in the final analysis. Patients required a stable medication with no change of dosage within the last six months. All patients had additional medication with different combinations of antiepileptic drugs (Table 1). MRI prior to study begin was performed to detect a nerve-vessel conflict and symptomatic causes for TN. None of the investigated patients had an invasive procedure prior to study inclusion (i.e., percutaneous ganglion gasseri procedures, microvascular decompression, gamma knife surgery). Patients with other chronic pain disorders or disorders of the nervous system were excluded. One patient additionally suffered from migraine. The local ethics committee of the medical faculty of the University of Duisburg-Essen approved the experimental protocol, all study participants provided written informed consent.

**Table 1:** Demographics and clinical characteristics

**Patient**	**Gender**	**Age (Years)**	**Localisation**	**VRS**	**AF**	**Medication**	**Duration of pain**	**Concomitant permanent pain**	**Nerve-vessel-conflict**	**Disease (Years)**
1*	f	68	V2 + V3; left	7	25	Doxepin/Carbamazepin	Min	YES	NO	6
2	f	77	V1; right	8	8	Gabapentin	Sec	NO	YES	14
3	f	53	V3; right	6	5	Carbamazepin/Pregabalin	Sec	NO	YES	20
4	f	50	V3; left	8	50	Oxcarbamazepin/Amitriptylin/Topamax	Sec	NO	NO	12
5	m	82	V1; left	8	3	Pregabalin/Duloxetin	Min	NO	NO	25
6	m	68	V3; right	3	5	Morphin/Pregabalin/	Min	NO	YES	20
7	f	78	V1; left	6	7	Amitriptylin/Tramal/Pregabalin	Min	YES	YES	15
8	f	65	V2 + V3; left	5	1	Carbamazepin/Pregabalin/Oxycodon	Min	YES	NO	21
9*	m	67	V2; right	5	1	Gabapentin/Oxcarbazepin/TrimipraMin	Min	NO	NO	4
10*	f	32	V2 + V3; left	8	15	Gabapentin/Oxcarbazepin	Min	NO	YES	8
11*	m	49	V2; right	7	8	Gabapentin/Fluoxetin	Min	YES	NO	27
12*	f	67	V2 + V3; right	7	7	Pregabalin/TrimipraMin	Min	YES	NO	2
13*	m	77	V2 + V3; left	5	4	Gabapentin/Sertralin	Sec	NO	YES	3
14*	f	34	V3; left	8	6	Gabapentin	Min	NO	YES	2
15*	f	52	V2 + V3; left	8	8	Carbamazepin/Oxycodon/Nortriptylin	Sec	YES	NO	8
16*	m	71	V3; right	5	4	Carbamazepin	Sec	NO	NO	7
17*	m	70	V2; left	4	5	Ibuprofen	Sec	NO	YES	27

VRS = verbal rating scale, AF = Attack frequency, *patients with complete data, that entered the final analysis.

### Pain assessment

Pain was measured using a verbal rating scale (VRS) as self-evaluation ranges from 0 (no pain) to 10 (maximal pain). VRS and attack frequency were assessed as an averaged value per day for one month before, during and after tDCS using an individual patient diary. The average value of three days prior to tDCS was used for baseline and the average of the last three days after day 14 (=post-stimulation) was used for further analysis. We used this to exclude a failure by single days with higher pain raitings which may occur in TN. Furthermore, participants documented the frequency of pain attacks per day in the same fashion and analysis was also based on the average of the last three days prior to assessment day (e.g., baseline and post-stimulation).

### Transcranial direct current stimulation

**Figure 1:**
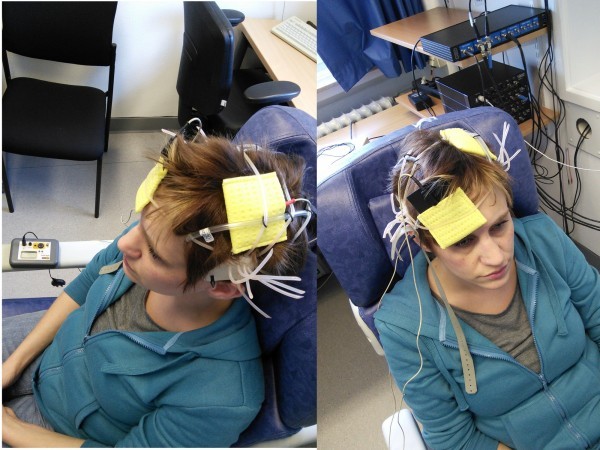
Fitting and configuration of the stimulation electrodes during transcranial direct current stimulation using a pair of surface rubber electrodes in a NaCl-solution soaked synthetic sponge over the primary motor cortex (M1) and above the contralateral orbit.

TDCS was delivered by a battery-driven constant current stimulator (Neuronica, Torino, Italy) with a maximum output of 5 mA using a pair of surface rubber electrodes in a NaCl-solution soaked synthetic sponge with an extension of 4×4 cm over the primary motor cortex (M1) and 5×10 cm above the contralateral orbit. The use of non-metallic rubber electrodes avoids electrochemical polarization (Figure 1). The electrode for tDCS is large, so that the stimulation encompassed a broad area of the motor cortex (upper limb and face). Patients received either anodal or sham stimulation, starting with either one in a double blind randomized fashion and were aware of the study design including sham stimulation. First, the hand area over the contralateral hemisphere to pain was determined by a single pulse transcranial magnetic stimulation (TMS). For anodal stimulation, the active electrode was placed over the hand representation field of the motor cortex and the reference was placed above the contralateral eyebrow according to the international 10–20 system for EEG electrode placement. The sham stimulation was administered by fixing the electrodes at the same positions and switching on the current for less than five seconds at a current strength below 500 μA in order to cause a slightly itching or tingling sensation and simulate the real current stimulation. Patients were asked if they feel the tingling. Thus, the patients felt the initial current sensation, but received no current for the rest of the stimulation period. With this procedure, patients cannot distinguish between verum and sham stimulation. The current was applied for 20 minutes at an intensity of 1.0 mA, according to current safety recommendations [17]. The maximum current density was 62.5 μA/cm^2^ over M1 and 12 μA/cm^2^ at the reference electrode. After the first stimulation, participants and at least one relative were instructed for the correct application of tDCS and were able to stimulate at home. Subsequently the patients applied the stimulation daily by themselves and were instructed to record daily, whether or not any potential side effects occurred in their diary. To ensure the correct application of tDCS, the stimulator records the correct current application in an electronic protocol. In cases of malfunctions or handling problems, the patients had the possibility to call the study team, which has not been used. Stimulation was performed for 14 days, 20 minutes per day. Following a cross-over study design participants were administered sham stimulation or anodal stimulation in a randomized order with an interval of at least one month after the first stimulation to avoid carry over effects.

### Electrophysiological settings

Two planar concentric electrodes (inomed Medizintechnik GmbH, Emmendingen, Germany, http://www.inomed.com) were attached to the skin 10 mm above the entry zone of the supraorbital nerve. The outer rim of the first electrode was placed 1 cm from the forehead midline, the second electrode approximately 2 cm apart and lateral. Left side and right side were stimulated 15 times per session in each patient (triple pulse, monopolar square wave, duration 0.5 ms, pulse interval 5 ms, interstimulus interval: 12 to 18 seconds, pseudo-randomized). Perception and pain thresholds were determined on the forehead with an ascending and descending sequence of 0.2 mA intensity steps. The stimulus intensity was set at 2 times the individual pain threshold. Stimuli were delivered to each side in random order in terms of start site (i.e., left or right side of the forehead). NBR and PREP were recorded simultaneously following trigeminal stimulation of the forehead. The nBR was recorded using surface electrodes placed infraorbitally referenced to the orbital rim. Recording parameters: bandwidth 1 Hz to 1 kHz, sampling rate 2.5 kHz, sweep length 300 ms (1401 plus, Signal, Cambridge Electronic Design, UK). PREP were recorded with electrodes placed at C_z_ referenced to linked earlobes (A1-A2) according to the international 10–20 system. The first sweep was rejected to avoid contamination by startle response. The remaining 14 sweeps were averaged. For nBR onset latencies, waveforms were rectified and analysed for each sweep separately. A mean value for each session was calculated. Area under the curve was calculated between 27-87 ms. Concerning PREP N2 (negative peak), P2 (positive peak) latencies and peak-to-peak amplitudes were analysed. Intraindividual mean ratios for latencies, amplitudes, and area under the curve were determined in all patients for the symptomatic and non-symptomatic pain side separately. Offline-analysis was performed with a custom-written PC-based software using Matlab (Matlab 7, The MathWorks, Natick, MA, USA).

### Statistical analysis

As of the small sample size non-parametric statistical methods were applied. Wilcoxon signed-rank test for paired samples was used to compare pain intensity (VRS), attack frequency, individual pain threshold, AUC, latency, and amplitude of electrophysiological experiments at baseline and after 14 days tDCS. Correlation analysis was performed investigating the association of electrophysiological response with clinical response to tDCS. All statistics were calculated with SPSS 22 (SPSS Inc., Chicago, IL, USA). The level of significance was set to p <0.05.

## Results

All patients tolerated tDCS well without adverse effects. With initiation of tDCS, patients feel a slight itching or tingling, while no motor symptoms were observed or reported during the stimulation. Six patients discontinued the trial prematurely as they did not have their expected treatment response. One patient did not stimulate at all. For characterization of patients see Table 1.

### Pain assessment after tDCS

**Figure 2:**
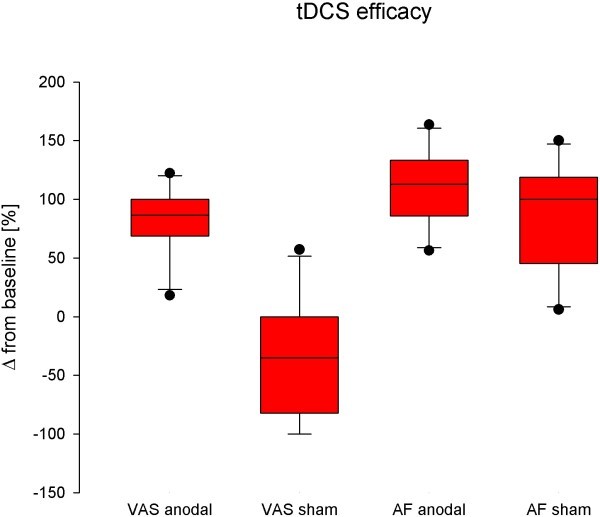
**Analgesic efficacy of anodal transcranial direct currents stimulation (tDCS).** tDCS significantly decreases mean pain intensity on the verbal rating scale (VRS) (p < 0.05), while attack frequency (AF) is not significantly different compared to control conditions. Changes of values are expressed as Δ compared to mean values under control conditions in box plots.

Pain intensity (VRS) difference comparing anodal stimulation effect (post-stimulation minus baseline) with the sham effect (post-stimulation minus baseline) was 29% (p = 0.008; Figure 2). VRS decreased after anodal stimulation from baseline by 18% (±SD 29%, while sham stimulation led to an 11% (±30.8%) increase of VRS (Figure 2, Table 1). Attack frequency was not significantly decreased between sham or anodal stimulation (p = 0.123); Figure 1). One patient was completely pain free after anodal stimulation.

In TN patients with purely paroxysmal pain tDCS decreases VRS by 24.2% after anodal stimulation and increased by 28% after sham stimulation (p = 0.032). Attack frequency decreases in either anodal or sham group not significantly (41.9 vs 56%; p = 0.148).

TN patients with concomitant persistent pain alone did not respond to tDCS at all (0%) change after anodal stimulation and 14.8% (SD ± 13.8%) after sham stimulation (p = 1.0). Attack frequency was not significantly different (p = 391; Table 2).

**Table 2:** Pain intensity and attack frequency

**All TN patients**	**Pain intensity (VRS)**	**Pain intensity (VRS)**	**Attack frequency/day**	**Attack frequency/day**
	**Baseline ± SD (range)**	**Post-stimulation ± SD (range)**	**Baseline ± SD (range)**	**Post-stimulation ± SD (range)**
**Anodal**	6.7 ± 1.3 (4.5-8)	5.5 ± 2.3 (1–8)	7.2 ± 7.9 (1–21)	4.9 ± 7 (0–22)
**Sham**	7.2 ± 1.2 (4.5-8)	7.8 ± 1.8 (4.5-10)	11.6 ± 15.1 (1–50)	6.7 ± 7.6 (1–21)
TN with concomitant permanent pain (n = 4*)				
**Anodal**	7.6 ± 0.3 (7.5-8)	7.6 ± 0.3 (7.5-8)	4.3 ± 6.5 (1–14)	6.3 ± 10.5 (1–22)
**Sham**	7.6 ± 0.3 (7.5-8)	8.8 ± 1.0 (7.5-10)	4.3 ± 6.5 (1–14)	6.0 ± 10 (1–21)
TN with purely paroxysmal pain (n = 6*)				
**Anodal**	6.1 ± 1.5 (4.5-8)	4.2 ± 1.8 (1–5.5)	9.2 ± 8.8 (1–21)	4.0 ± 4.6 (0–12)
**Sham**	7.0 ± 1.6 (4.5-8)	7.2 ± 2.0 (4.5-10)	16.5 ± 17.7 (1–50)	7.1 ± 6.7 (1–16)

VRS = verbal rating scale (0 = no pain; 10 = maximum pain); SD = standard deviation.

*only patients that entered the final analysis.

### PREP and nBR after tDCS

**Figure 3:**
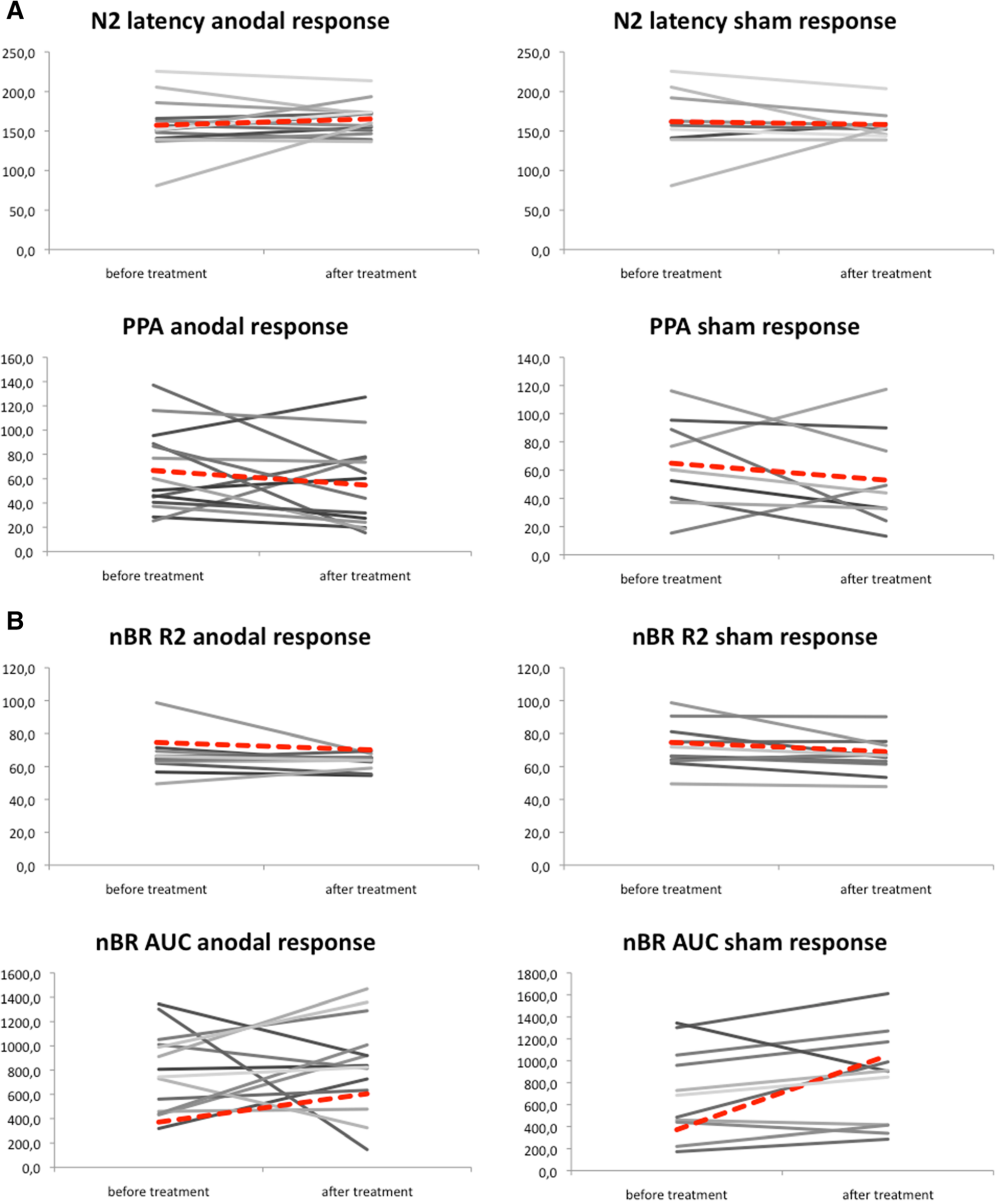
**Effects of anodal transcranial direct current stimulation (tDCS) on (A) pain-related evoked potentials (PREPs) and (B) nociceptive blink reflex (nBR).** Anodal tDCS results in decreased trigeminal peak-to-peak amplitudes and increased N2 latencies compared to baseline. Trigeminal N2 latencies and area under the curve are not significantly different between anodal tDCS and sham stimulation. Grey lines mark single patients, red lines mark mean values.

Trigeminal PREPs and nBR were elicited using similar current intensities without significant differences between patients after both, anodal tDCS and sham stimulation, compared to baseline conditions (Figure 3, Table 3). No relevant changes were apparent in nBR R2 latencies and nBR areas under the curve (Figure 3B, Table 3) so that brainstem trigeminal nociceptive processing is not equally affected by tDCS. There were no relevant differences in PREP or nBR between patients with and without concomitant permanent pain or in comparison of symptomatic and asymptomatic side. No correlation of PREP or nBR with VRS or attack frequency was found.

**Table 3:** Electrophysiological data – nociceptive blink reflex and pain related evoked potentials

Nociceptive Blink reflex*	**Condition**	**Latency (ms)**	**Latency (ms)**	**Area under the curve (x10**^**-3**^**, μVxms)**	**Area under the curve (x10**^**-3**^**, μVxms)**
		***Baseline***	***Post-stimulation***	***Baseline***	***Post-stimulation***
**Symptomatic side**	anodal	66.6 ± 11.0	63.8 ± 4.6	766.3 ± 345.3	917.9 ± 363.9
	Sham	71.9 ± 14.0	66.7 ± 11.2	685.1 ± 418.9	851.2 ± 434.4
**Asymptomatic side**	anodal	65.5 ± 7.9	74.4 ± 47.1	863.1 ± 404.7	827.7 ± 380.4
	Sham	66.6 ± 7.1	64.6 ± 8.1	819.2 ± 449.1	839.3 ± 524.4
Pain related evoked potentials*	**Condition**	**Amplitude (μV)**	**Amplitude (μV)**	**N2 latency (ms)**	**N2 latency (ms)**
		***Baseline***	***Post-stimulation***	***Baseline***	***Post-stimulation***
**Symptomatic side**	anodal	66.7 ± 34.1	54.8 ± 34.9	157.4 ± 34	165.4 ± 20.9
	Sham	58.5 ± 32.4	48.9 ± 31.8	162.5 ± 38.3	163.3 ± 20.7
**Asymptomatic side**	anodal	55.3 ± 31	55.9 ± 30.8	155.6 ± 24.2	169.7 ± 48.8
	Sham	50.8 ± 28.1	52.7 ± 35.6	161.1 ± 22.5	161.4 ± 23.9

AUC = Area under the curve; *only patients that entered the final analysis.

## Discussion

Our results provide evidence that anodal tDCS of the primary motor cortex (M1 area) contralateral to the symptomatic side moderately ameliorates pain in patients with classical TN with purely paroxysmal pain by modulation of trigeminal nociceptive processing. Patients with concomitant permanent pain do not seem to benefit from tDCS [18-21].

To our knowledge, the effects of anodal tDCS on facial pain were investigated in only one study, including 22 patients with different pain syndromes. Three patients suffered from TN and one patient from persistent idiopathic facial pain. However, the efficacy of pain relief in these patients was not analyzed separately, so that a clear effect on TN specifically cannot be derived from these data [6]. Comparing our data with previous studies that used anodal tDCS for the treatment of pain we found a slightly attenuated analgesic effect with a reduction of VRS by 29% compared to a maximum of 50% that was previously described [6,22]. This increases to 38% when we remove those patients with concomitant permanent pain from the analysis that did not respond to tDCS, but remains in a rather moderate nevertheless realistic range.

TDCS has been used more and more frequently over the past decade as a non-invasive neuromodulation for targeted alteration of cortical excitability for the treatment of different diseases like depression, reorganization after stroke and pain [23-25]. Effects of tDCS depend on several factors including polarity, stimulated area, intensity and duration. The improvement of pain by anodal tDCS was convincingly demonstrated in several studies [6,22,11]. Although the exact mechanism behind the efficacy of tDCS on chronic pain is not known, probable mechanisms are the changes of the resting membrane potential under the active electrode and remote effects in other parts of the pain processing network by functional interconnections between motor-cortex driven inhibition of the somatosensory cortex and changes in thalamic activity. In addition, long-term effects are mediated by modulation of i.e. NMDA and nicotinic receptor activity inducing neuroplastic effects [5]. Furthermore, anodal stimulation was shown to induce an increase of endogenous opioid release [26] which was located in parts of the pain processing network after tDCS over the M1 area.

When pain becomes chronic tDCS modulation might be less prominent [14]. This could explain why TN patients with concomitant permanent pain in our study did not respond. Moreover, it was demonstrated that the analgesic effect of tDCS critically depends on the electrode montage. An optimized cortical target supported by high-resolution computational models including CNS structures of the pain processing network markedly improved tDCS efficacy [27] and utilization of smaller, more focal electrodes led to significant pain reduction in fibromyalgia [28]. Differences in electrode montage might explain some of the contradictory results in different studies.

Central facilitation and hyperexcitability of the trigeminal system was recently demonstrated in TN patients using PREP and nBR [19]. Other reports presented an increased fMRI activation and grey matter changes of cortical pain processing networks to further support an involvement of cortical structures [29,21]. It was shown that tDCS affects human trigeminal pain processing [30]. Recordings of PREP and nBR before and after anodal tDCS resulted in decreased peak-to-peak amplitudes of PREP suggesting an inhibition of trigeminal pain processing which provides evidence that tDCS is a potential therapeutic option in disorders associated with central facilitation. Anodal tDCS modulates pain processing by inhibition of corticothalamic epicritic and nociceptive sensations at the thalamic nuclei [31] and induces changes in brain synchronization in the stimulated area [32]. Moreover, changes were observed in the anterior cingulate cortex which was suggested to be the generator of PREP in humans [33]. These observations reconfirm tDCS to interfere with TN pathophysiology. The results in the anodal group of our study showed an inhibition of trigeminal pain processing after tDCS in PREP similar to what was observed previously in healthy patients [30] even though these changes did not reach the level of significance due to the small sample size.

Supposing that the intensity of TN pain depends on combined peripheral and central mechanisms, the attack frequency may be more dependent on peripheral mechanisms e.g. nerve-vessel contact as trigger and, therefore, may not be equally well modulated by cortical stimulation. This might explain why the tDCS effect on attack frequency was insignificant.

The limitations of this study have to be considered. The relatively high drop out rate with more female than male patients shows that self-tDCS application at home may pose a huge problem for the mostly elderly TN target population, even though side effects were not reported and tolerability of participating patients was high. The stimulation with tDCS appears to be more difficult for the patients than previously suspected and probably requires a considerable education effort before it can be used sufficiently. Stimulation frequency (every day for 14 days) may not be optimal as a shorter duration may be just as effective or longer duration may be even more effective, but that will have to be tested in future studies. Due to the enormous interindividual variability in regard to tDCS treatment response it might be difficult to find the optimal stimulation parameters that suits all patients.

All patients received therapy with different anti-epileptic medications that may have influenced clinical and electrophysiological data. We tried to minimize this impact by requiring a stable dose for six months prior to study inclusion. Carry-over effects due to the cross over trial design must be considered despite the 30-day interval between treatment conditions. There is evidence that patients with a special polymorphism in the BDNF receptor gene showed a pronounced facilitation after anodal and inhibition after cathodal tDCS [34]. This may also have affected our patients, but due to the low patient numbers we did not want to exclude anybody and, therefore, did not control for this. Long term effects of tDCS have to be considered. The duration of tDCS effects depend on several factors including the stimulation protocol, remote effects of other cortical areas and modulation of neuronal receptor activity beside NMDA-R effects. These factors are highly individual and underline the complexity of tDCS effects. It has been shown that brain plasticity depends also on the modulation of nicotinic receptors, BDNF polymorphisms and sex hormonal variations [35,36]. Therefore, the differences in efficacy of the acute and long-term effects of tDCS may depend on factors that are hard to control in studies with rare diseases and low patient numbers.

## Conclusion

In summary, tDCS is a promising non-invasive treatment option for trigeminal neuralgia without side effects in patients without satisfying response to medical treatment. Nonetheless, larger studies are needed to clarify the mechanism of action of tDCS in trigeminal pain and to reconfirm its efficacy in TN.

## Competing interests

Tim Hagenacker has received research support from Astellas and CSL Behring.

Vera Bude, Steffen Naegel have nothing to disclose.

Dagny Holle has received research support from Grünental and Allergan.

Mark Obermann has received scientific support and/or honoraria from Biogen Idec, Novartis, Sanofi-Aventis, Genzyme, Pfizer, Teva. He received research grants from Allergan, Electrocore, and the German Ministry for Education and Research (BMBF).

Hans-Christoph Diener has received honoraria for participation in clinical trials, contribution to advisory boards or lectures from Addex Pharma, Allergan, Almirall, AstraZeneca, Bayer Vital, Berlin Chemie, Coherex Medical, CoLucid, Böhringer Ingelheim, Bristol-Myers Squibb, GlaxoSmithKline, Grünenthal, Janssen-Cilag, Lilly, La Roche, 3M Medica , Minster, MSD, Novartis, Johnson & Johnson, Pierre Fabre, Pfizer, Schaper and Brümmer, SanofiAventis, and Weber & Weber; received research support from Allergan, Almirall, AstraZeneca, Bayer, Galaxo-Smith-Kline, Janssen-Cilag, and Pfizer. Headache research at the Department of Neurology in Essen is supported by the German Research Council (DFG), the German Ministry of Education and Research (BMBF), and the European Union.

## Authors’ contributions

MO and ZK conceptualized the experimental design. MO and TH organized the study. TH and VB acquired the data. TH, VB, SN and DH were responsible for the clinical supervision of the test persons. TH, DH, HD and MO analyzed the data. MO conducted the statistical analysis. TH wrote the first draft of the manuscript and interpreted the findings. All authors gave input to the manuscript. All authors read and approved the final manuscript.
